# Isolation of *Tibet orbivirus*, TIBOV, from *Culicoides* Collected in Yunnan, China

**DOI:** 10.1371/journal.pone.0136257

**Published:** 2015-08-21

**Authors:** Wenwen Lei, Xiaofang Guo, Shihong Fu, Yun Feng, Kai Nie, Jingdong Song, Yang Li, Xuejun Ma, Guodong Liang, Hongning Zhou

**Affiliations:** 1 State Key Laboratory for Infectious Disease Prevention and Control, National Institute for Viral Disease Control and Prevention, Chinese Center for Disease Control and Prevention, Beijing, People’s Republic of China; 2 Collaborative Innovation Center for Diagnosis and Treatment of Infectious Diseases, Hangzhou, People’s Republic of China; 3 Yunnan Institute of Parasitic Diseases, Pu’er, Yunnan, People’s Republic of China; 4 Yunnan Institute of Endemic Disease Control and Prevention, Yunnan Provincial Center of Virus and Rickettsia Research, Dali, Yunnan, People’s Republic of China; University of Texas Medical Branch, UNITED STATES

## Abstract

We isolated a novel virus strain (YN12246) from *Culicoides spp*. specimens collected at the China-Laos-Myanmar border in southern Yunnan Province. This virus had a cytopathic effect (CPE) on both insect cells (C6/36) and mammalian cells (BHK-21). Electron microscopy revealed the structure of the virions to be spherical with a diameter of 75 nm. Polyacrylamide gel analysis demonstrated that the viral genome consisted of 10 segments of double-stranded RNA (dsRNA), with a distribution pattern of 3-3-3-1. The coding sequences of 9 genome segments of YN12246 (Seg1, Seg3-Seg10) were obtained by high-throughput sequencing and Sanger sequencing. Comparisons of conserved genome segments 1 and 3 (Seg1 and Seg3), encoding the polymerase-VP1 and sub-core T2 protein, respectively, showed that YN12246 groups with the *Culicoides*-borne orbiviruses. The highest levels of sequence identity were detected between YN12246 and *Tibet orbivirus* (TIBOV), indicating that they belong to the same virus species (with amino acid identity of 98.8% and 96.4% for the polymerase and T2 protein, respectively). The data presented here confirm that YN12246 is a member of the TIBOV species, which was first isolated from mosquitoes in 2009. This is the first report of the isolation of TIBOV from *Culicoides*.

## Introduction

The genus *Orbivirus* is the largest of 15 genera within the family *Reoviridae*, currently containing 22 recognized virus species [1]. Several species are known to cause serious disease in animals, and orbivirus infections have also been documented in association with human disease [2]. The orbiviruses are vector-borne, primarily transmitted by ticks or hematophagus insect-vectors (including *Culicoides*, mosquitoes and sand flies), and have a wide host-range that collectively includes domestic and wild ruminants, equines, marsupials, sloths, bats, birds and humans [3–5]. Previous research has shown that oribiviruses can be divided into three groups, namely tick-borne, mosquito-borne and *Culicoides*-borne, according to evolutionary data [1]. *Bluetongue virus* (BTV), *African horse sickness virus* (AHSV) and *Epizootic hemorrhagic disease virus* (EHDV) are transmitted by adult *Culicoides* and are regarded to be the most important orbiviruses with respect to economics [3, 6]. Although sequence data are available for several of the insect-borne orbiviruses, few of the tick-borne orbivirus species have been analyzed, including *Broadhaven virus* (BRDV; a Great Island virus species) [7] and *St*. *Croix River virus* (SCRV; a St. Croix River virus species) [8]. Comparison of the sequences of homologous proteins has revealed greater genetic divergence between the insect-borne and tick-borne orbiviruses (with 23–38% amino acid (aa) identity) than among the species within either of these groups [8].

The *Tibet orbivirus* (TIBOV) was first isolated by our laboratory from *Anopheles maculatus* mosquitoes collected in 2009 from Motuo County in Nyingchi Prefecture, Tibet, China. Molecular genetic analysis revealed that this virus was a new species within the genus *Orbivirus* [9], denoted the *Tibet orbivirus*. Although this virus was first isolated from mosquito specimens, molecular evolutionary genetics analysis revealed that it was not closely related to the mosquito-borne orbiviruses, and was instead a better fit for the *Culicoides*-borne group. Because only one strain (XZ0906) was available for analysis, it was difficult to determine the propagation characteristics of TIBOV.

During a survey of arboviruses in Yunnan Province in 2012, our laboratory isolated a virus strain (YN12246) from a pool of *Culicoides spp*. specimens, and identified it as a TIBOV strain. Here, we describe the sequence analysis of polymerase (Pol) and T2-subcore-protein of TIBOV. Phylogenetic comparisons with different orbiviruses indicate that TIBOV belongs to the *Culicoides*-borne orbivirus group. This is the first report of TIBOV isolated from *Culicoides*.

## Materials and Methods

### 1. Cell culture

C6/36 *Aedes albopictus* cells and BHK-21 baby hamster kidney cells were used in this study [10]. Both cell lines were maintained in our laboratory. C6/36 cells were cultured in 45% DMEM, 45% RMPI 1640 (Invitrogen), 10% fetal bovine serum (FBS; Invitrogen) and 100 U/mL of penicillin and streptomycin. C6/36 cells were propagated and maintained at 28°C. BHK-21 cells were cultured in Eagle's medium containing 10% FBS (Invitrogen), 2 mM glutamine, 0.12% NaHCO_3_ and 100 U/mL of penicillin and streptomycin. BHK-21 cells were maintained at 37°C in the presence of 5% CO_2_.

### 2. Sample collection and virus isolation

*Culicoides* samples were collected in the summer of 2012 in Daluo, Xishuangbanna Dai Autonomous Prefecture, Yunnan province, China. The specimens were frozen to death in a -20°C freezer, then classified and identified on ice, dispensed into cell-freezing tubes, recorded and labeled, stored in liquid nitrogen and finally transported to our laboratory. Specimens were then homogenized, inoculated and blind passaged three times (7 days per cycle) on monolayers of C6/36 and BHK-21 cells in 24-well plates, as previously described [11]. The cells were observed daily for signs of a cytopathic effect (CPE). The culture supernatant was stored at -80°C for further analysis.

### 3. Electron microscopy of virions

The culture supernatant from infected BHK-21 cells showing signs of CPE was centrifuged at 8000 rpm for 20 min at 4°C. The resulting supernatant was further centrifuged at 30,000 rpm for 2 h to remove cellular debris. The final supernatant was fixed in 2% glutaraldehyde and processed for negative-stain electron microscopy with 2% phosphotungstic acid.

### 4. dsRNA-polyacrylamide gel electrophoresis

Viral RNA was isolated as described previously, and analyzed by polyacrylamide gel electrophoresis (PAGE) [12].

### 5. Virus identification by RT-PCR

Viral RNA was extracted using the QIAamp Viral RNA Mini Kit (Qiagen), and cDNA was synthesized using Ready-To-Go You-Prime First Strand Beads (GE Healthcare), according to the manufacturer’s procedure. Samples were subjected to Reverse-transcription polymerase chain reaction (RT-PCR) with specific primers designed to detect the 12th segment of Banna virus (BAV) [13], the 12th segment of Liao ning virus (LNV) [14], the 12th segment of Kadipiro virus (KDV) [15], the 7th segment of Yunnan orbivirus (YUOV) [16] and the 1st and 3rd segments of *Tibet orbivirus* (TIBOV) [9] (Table 1). Positive samples were sequenced in both directions and the obtained sequences were compared with those available in public databases (GenBank).

**Table 1:** Primers used in this study to identify viruses.

Virus	Primerdesignation	Orientation	Sequence(5’-3’)	Size	Reference
BAV	12-854-S	Sense	AAATTGATAGYGYTTGCGTAAGAG	845	13
	12-B2-R	Antisense	GTTCTAAATTGGATACGGCGTGC		
LNV	LNV12s1	Sense	GGAAGAATCAATGCCGTAGCCAC	482	14
	LNV12r1	Antisense	GTGACGATCTTCTCTGAACCAGTG		
KDV	KDV 12F	Sense	GACGCTTTGAGATTATCTCGAC	758	15
	KDV 12R	Antisense	GCTCAATCGCATTCTCACC		
YUOV	YUOVS7F	Sense	AGCATTCGGTACGCAGTATCTCG	470	16
	YUOVS7R	Antisense	GCCGAGCCGATCATGTCACGTGT		
TIBOV	6-1-2F	Sense	TGGAGGAAGAGGGCGTGAG	869	9
	6-1-2R	Antisense	TAGAACCCTTTGTTTGGT		
	6-3-4F	Sense	TATTGGAGCGTGAAGCAT	713	9
	6-3-4R	Antisense	GTAAGTGTATTCCCGTTGCAGTCGG		

**Note:** BAV = Bannan virus; LNV = Liao ning virus; KDV = Kadipiro virus; YUOV = Yunnan orbivirus; TIBOV = Tibet orbivirus.

### 6. Preparation of viral DNA and RNA and Ion Torrent sequencing

Viral DNA was extracted from 200-μL aliquots of virus-infected BHK-21 cell culture supernatants using a QIAamp DNA BloodMini Kit (Qiagen). Viral RNA was extracted from 140-μL aliquots of virus-infected BHK-21 cell culture supernatant using a QIAamp Viral RNA Mini Kit (Qiagen) according to the manufacturer’s instructions. cDNA was made with a Ready-To-Go kit (GE Healthcare) using random hexanucleotide primers. Samples were then amplified as described previously [9]. Amplification products were sequenced at the National Institute for Viral Disease Control and Prevention, Chinese Center for Disease Control and Prevention. The Ion Sequencing Kit (Life Technologies) was used with the Personal Genome Machine (PGM) sequencer as described in the Ion Sequencing Kit User Guide [17] (part no. 4467391 rev. B, 04/2011).

RT-PCR was performed to fill in gaps between viral gene sequences obtained with Ion Torrent sequencing using contig-specific primers. Total viral RNA was extracted as described above, cDNA was generated by reverse transcription, and used as a template for complete genome amplification. Next, a set of specific primers was designed to amplify each segment of the viral genome and the amplification products were sequenced using the Sanger method (Table 2).

**Table 2:** Primers used in this study.

Primer	Position^a^	Sequence(5’-3’)	Orientation
1-1F	1–20	AACTGTGCAAGGCGCAGATC	Sense
1-1R	812–833	CGACTATAGGGTTAGCTGCAAC	Antisense
1-2F	677–696	GCGCTCGAGTTCGGTGAAAC	Sense
1-2R	1419–1441	GATAACCAACGCTTTGATTCGTG	Antisense
1-3F	1216–1237	GGTATCTGATTGTACACGCGAG	Sense
1-3R	2093–2112	GCCACGAGCATATCGAAACC	Antisense
1-4F	1847–1865	GCGATGGAGCCGTACAAAC	Sense
1-4R	2611–2629	CGCTCCTACAAACGCGGAC	Antisense
1-5F	2458–2479	CATACCTCAAGACCGTATGATG	Sense
1-5R	3289–3308	GTATGTAGAACTGCGGTGAC	Antisense
1-6F	3057–3078	CTCGGACTATGATGCATAGCGC	Sense
1-6R	3887–3911	GCGCTAATTAAACGAACTGATTCTC	Antisense
3-1F	1–25	GTAAAATTTCCGTGGCGATGGCTGA	Sense
3-1R	814–835	GGATTAATCGGGAGCTGCAGCG	Antisense
3-2F	631–650	GCGCATGTTCTGAACCGGTG	Sense
3-2R	1410–1433	GTATCGAAGAAATCGCTGCACGTG	Antisense
3-3F	1270–1293	CGACTGGTGAGCCGTTAGATTTCC	Sense
3-3R	2055–2074	CACGCTTCGCGTTCCAGAAC	Antisense
3-4F	1872–1891	CAGCGACGTGCACCAGATAC	Sense
3-4R	2673–2693	GGTGCCCATCTTGAGCGCTTG	Antisense
4-1F	1–25	GTAAAAACATGCCGGAGCCACATGC	Sense
4-1R	819–841	CACTCATCATCCGTCTGTTCGCC	Antisense
4-2F	633–653	CAGAGGAGCGAAGGTCGGTTT	Sense
4-2R	1392–1412	ATCCATCACGCGCACATCACG	Antisense
4-3F	1171–1194	GGCTGCACATCGAACATGTGAATG	Sense
4-3R	1952–1978	GTAAGTGTAACATGCCTTCCAGATCCG	Antisense
5-1F	1–25	GTAAAAAAGTTCTTCGTCGACTGCC	Sense
5-1R	800–820	TGTCTGCATCGTAAGCCTTGC	Antisense
5-2F	650–670	GACAGTGGATTTCAGGGACCG	Sense
5-2R	1430–1452	CCATCATGTCTTGGTTCATAGCC	Antisense
5-3F	1259–1282	GCATTGATTACGACAGCAATCACG	Sense
5-3R	1749–1775	GTAAGTGTAAGTTYGATAGAGCGAAGC	Antisense
6-1F	1–28	ATGCAAGAAGAAGCAATACAAGAAATTG	Sense
6-1R	950–974	AAATCTAATTACGCTGCAACCACTC	Antisense
7-1F	1–25	GTAAAAATTTGGTGAAGATGGACGC	Sense
7-1R	696–719	GTGCACAGTGATTGGATTCATTCC	Antisense
7-2F	515–537	CGCAAGAGGTGATATACAGGGTG	Sense
7-2R	1137–1163	AAGTGTAATTTGGGAAAACGTATGAAC	Antisense
8-1F	1–25	TAAAAAAATTCCTAGCAACCATGGA	Sense
8-1R	661–681	CTCGGTTCCAAACGTCCCATC	Antisense
8-2F	462–481	GTCCGAATCGCAATACGATG	Sense
8-2R	1122–1143	GTAAGTTTGAATTCCCTCCCCT	Antisense
9-1F	1–25	GTAAAAAATTGCTTATGTCAGCTGC	Sense
9-1R	647–669	CTATGTAGATGACGTTGAGTGAC	Antisense
9-2F	457–474	GCACTGAACTTGCTGCGG	Sense
9-2R	1075–1100	GTAAGTTTTGAATTGCTACGGTCAGG	Antisense
10-1F	1–25	GTAAAAAAGAGTGTGGTTGTCATGC	Sense
10-1R	814–833	GTAAGTTGGCGGGAATGCGG	Antisense

^a^ Position refer to the genome of XZ0906 (GenBank accession number KF746187 to KF746196).

### 7. Phylogenetic analysis

Multiple sequence alignments were performed using the Clustal X 1.8 software. Phylogenetic and molecular evolutionary analyses were conducted using the Mega 5.05 software. The phylogenetic tree was generated using the neighbor-joining (NJ) method, distance-p model and 1000 replications of bootstrap values. The sequences obtained in this study were compared with those available in public databases, and representative *Reoviridae* and *Orbivirus* sequences were used for phylogenetic analysis. The background information of all virus strains used in this study is shown in S1 Table.

## Results

### 1. Virus isolation

**Fig 1:**
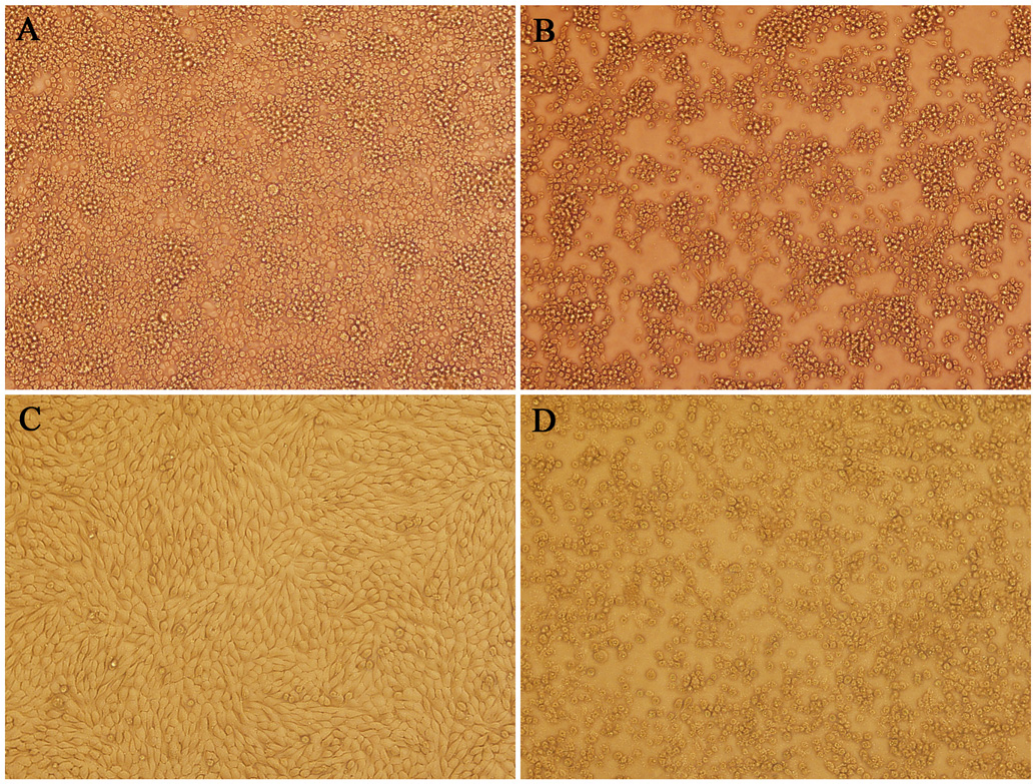
Cytopathic effect of YN12246 on C6/36 and BHK-21 cells (× 200). (A) Uninfected C6/36 control cells (96 h). (B) Infected C6/36 cells, 96 h post-infection, showing cell rounding, aggregation and detachment of infected cells. (C) Uninfected BHK-21 control cells (72 h). (D) Infected BHK-21 cells, 72 h post-infection, showing cell rounding and rupture.

A single pool of *Culicoides spp*. homogenized supernatants was inoculated onto monolayers of C6/36 and BHK-21 cells, yielding a virus isolate designated YN12246. The supernatant of YN12246 resulted in CPE in C6/36 and BHK-21 cells in successive cell passages. By 48 h post-infection, shrinkage, aggregation and shedding were observed in C6/36 cultures, and rounding, lysis and shedding were observed in BHK-21 cells (Fig 1).

### 2. Observation of virus morphology

**Fig 2:**
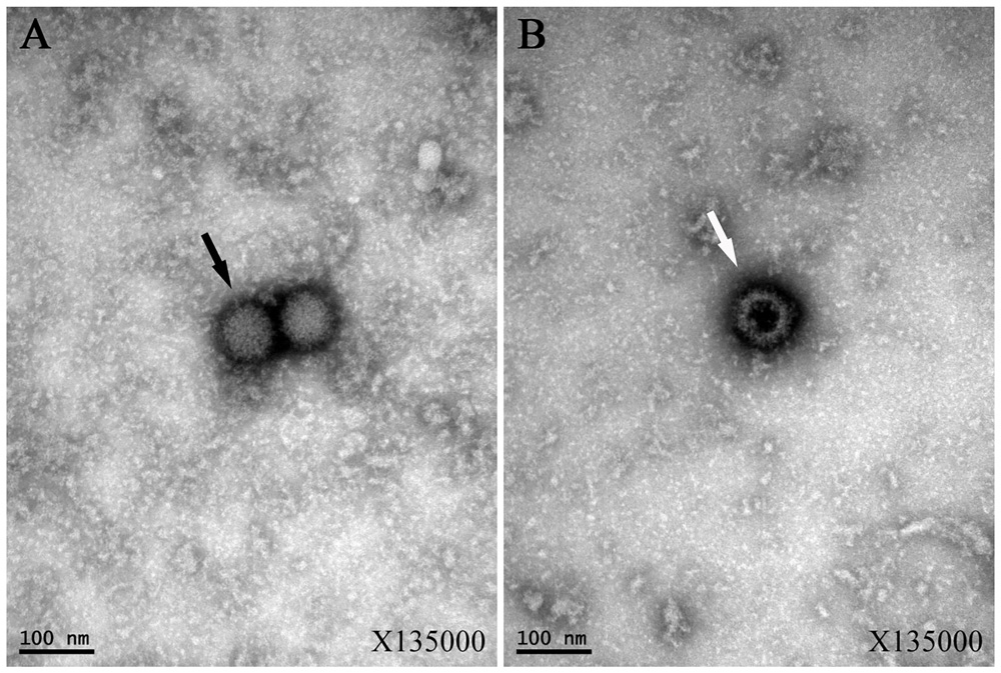
Electron micrographs of YN12246 particles negatively stained with 2% potassium phosphotungstate. (A) The black arrow indicates an intact particle; (B) The white arrow indicates an empty particle.

The supernatant of cultures showing signs of CPE after virus infection were subjected to negative staining with phosphotungstic acid. Using transmission electron microscopy, YN12246 particles were observed to be approximately 75 nm in diameter, spherical, lacking an envelope, and with an obvious capsomere on the particle surface (Fig 2).

### 3. Identification of the dsRNA genome structure

**Fig 3:**
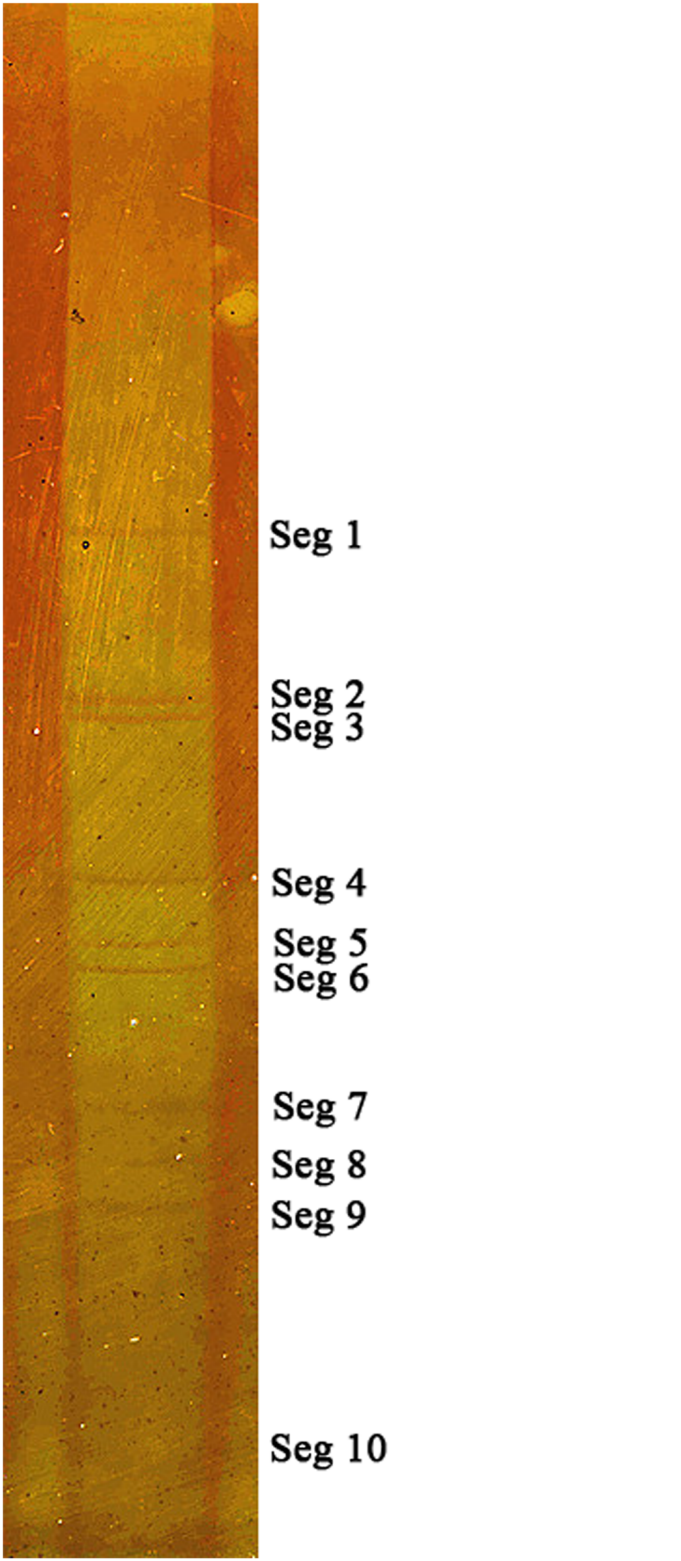
Electrophoretic migration patterns of YN12246 dsRNA. Standard discontinuous polyacrylamide gel electrophoresis was used, with a 3.5% acrylamide concentration gel and 10% acrylamide separation gel. After staining with silver nitrate, the genome of YN12246 was visualized as 10 distinct bands.

Viral RNA was harvested from the culture supernatant of infected BHK-21 cells, and analyzed by PAGE, revealing a 10-segmented double-stranded RNA genome with a migration pattern of 3-3-3-1. Within this pattern, segments 7, 8 and 9 were very weak, but still identifiable, whereas segment 10 was extremely weak (Fig 3).

### 4. Virus identification

RT-PCR, using primers designed to target Seg1 (869 bp long) and Seg3 (713 bp long) of the TIBOV genome, generated cDNA of the expected sizes (885 bp for Seg1 and 706 bp for Seg3) from the YN12246 template. However, reactions using primers designed to amplify BAV, LNV, KDV and YUOV all failed to generate PCR amplicons. Sequence analysis showed that Seg1 and Seg3 from YN12246 has a high level of nucleotide sequence identity (94% and 80%, respectively), and a high level of aa identity (98% and 95%, respectively), with TIBOV. Sequence analyses of Seg1 and Seg3 and comparisons with published data, demonstrated that the YN12246 virus from Yunnan is a strain of TIBOV.

### 5. Sequence analysis of the TIBOV genome segments

The whole coding sequences of 8 segments of YN12246 (Seg1, Seg3-Seg5, Seg7-Seg10) and partial sequences of Seg6 were obtained by high-throughput sequencing and Sanger sequencing. Differences were observed in both the nucleotide and amino acid sequences of virus YN12246 relative to XZ0906 (Table 3). The results showed that there are only 4 segments of YN12246 are the same as the mosquito-isolated strain (XZ0906) among the 8 segments available in the nucleotides length of the complete coding sequence. The identical ones are Seg-4, 5, 7 and 9 respectively, and the rest segments are shorter than that of the mosquito-isolated strain (XZ0906). As shown in Table 3, the difference of the nucleotides length between *Culicoides*-isolated strain (YN12246) and mosquito-isolated strain (XZ0906) is among 9–135 bp, the difference between Seg1 and Seg8 is above 100 bp, the difference between Seg3 and Seg10 is within 100 bp. It means that there is a big difference in the genome length among TIBOV isolated from different vectors. Besides, there is a larger difference (63.6–99.6%) in the homologous aa with each segment between *Culicoides*-isolated strain (YN12246) and mosquito-isolated strain (XZ0906). Seg1, 3, 4, 5, 7 and 9 of YN12246 exhibited a relatively high aa identity (95.1–99.6%) to their homologues in the XZ0906. Seg8 and 10 are slightly lower than that aa sequence identity respectively for 81.1% and 74%, and the identity between YN12246 and XZ0906 is 63.6% in Seg 6 (partial sequence).

**Table 3:** Comparison of coding sequence between *Culicoides*-isolated strain (YN12246) and mosquito-isolated strain (XZ0906) of TIBOV virus.

Segment	XZ0906		YN12246		Identity of amino acid (%)
	Length (bp)	Protein (aa)	Length (bp)	Protein (aa)	
S1	3915	1304	3780	1259	98.8
S3	2700	899	2676	891	96.4
S4	1932	643	1932	643	99.1
S5	1665	554	1665	554	99.6
S6	1581	526	951	316	63.6
S7	1050	349	1050	349	99.4
S8	1080	359	957	318	81.1
S9	1041	346	1041	346	95.1
S10	705	234	696	231	74

**Note:** Seg1, 3–5, 7–10 of YN12246 are the whole coding sequences, and Seg6 is the partial coding sequence (5’→3’: 660–1633).

The sequences of Seg2 and partial sequences of Seg6 of YN12246 were not obtained by high throughput sequencing and gene specific amplification (see the discussion section). The 1003 bp nucleotide sequences (660–1633) of Seg6 of YN12246 were obtained, and the rest sequences of Seg6 of YN12246 were not obtained (see Table 3). The sequence data were deposited into the GenBank database (accession numbers KP099640 and KP099641 for Seg1 and Seg3, respectively).

Whole genome sequence information of representative members of genus *Orbivirus*, including BTV, EHDV, YUOV, *Pata virus* (PATV), *Palyam virus* (PALV), and SCRV, was obtained from NCBI GenBank. The Seg1 and Seg3 sequences were downloaded and compared with those of YN12246.

Amino acid sequence analysis of VP1 protein (RNA-dependent RNA polymerase, RdRp), encoded by Seg1, revealed 98.8% sequence similarity between YN12246 and the TIBOV strain XZ0906, compared to between 35.4% (SCRV) and 73% (PATAV) similarity with the six representative orbiviruses. In the case of T2 protein, encoded by Seg3, sequence similarity between YN12246 and the TIBOV strain XZ0906 was of 96.4%, compared to between 23.3% (SCRV) and 78.3% (PATAV) similarity with the six representative orbiviruses (Table 4).

**Table 4:** Comparison of VP1 and T2 nucleotide numbers and amino acid identities between YN12246 and representative orbiviruses.

Protein	TIBOV		BTV-6		EHDV-6		PATAV		PALV		SCRV		YUOV	
	nt	aa(%)	nt	aa(%)	nt	aa(%)	nt	aa(%)	nt	aa(%)	nt	aa(%)	nt	aa(%)
VP1	3950	1304(98.8)	3944	1302(71.5)	3942	1302(72.4)	3948	1303(73)	3930	1295(59.5)	4089	1345(35.4)	3993	1315(47.7)
T2	2769	899 (96.4)	2772	901(77)	2768	899(77.5)	2770	899(78.3)	2774	904(57.7)	2747	890(23.3)	2900	940(38.3)

**Note:** TIBOV is the prototype isolate of XZ0906.

### 6. Phylogenetic analysis

**Fig 4:**
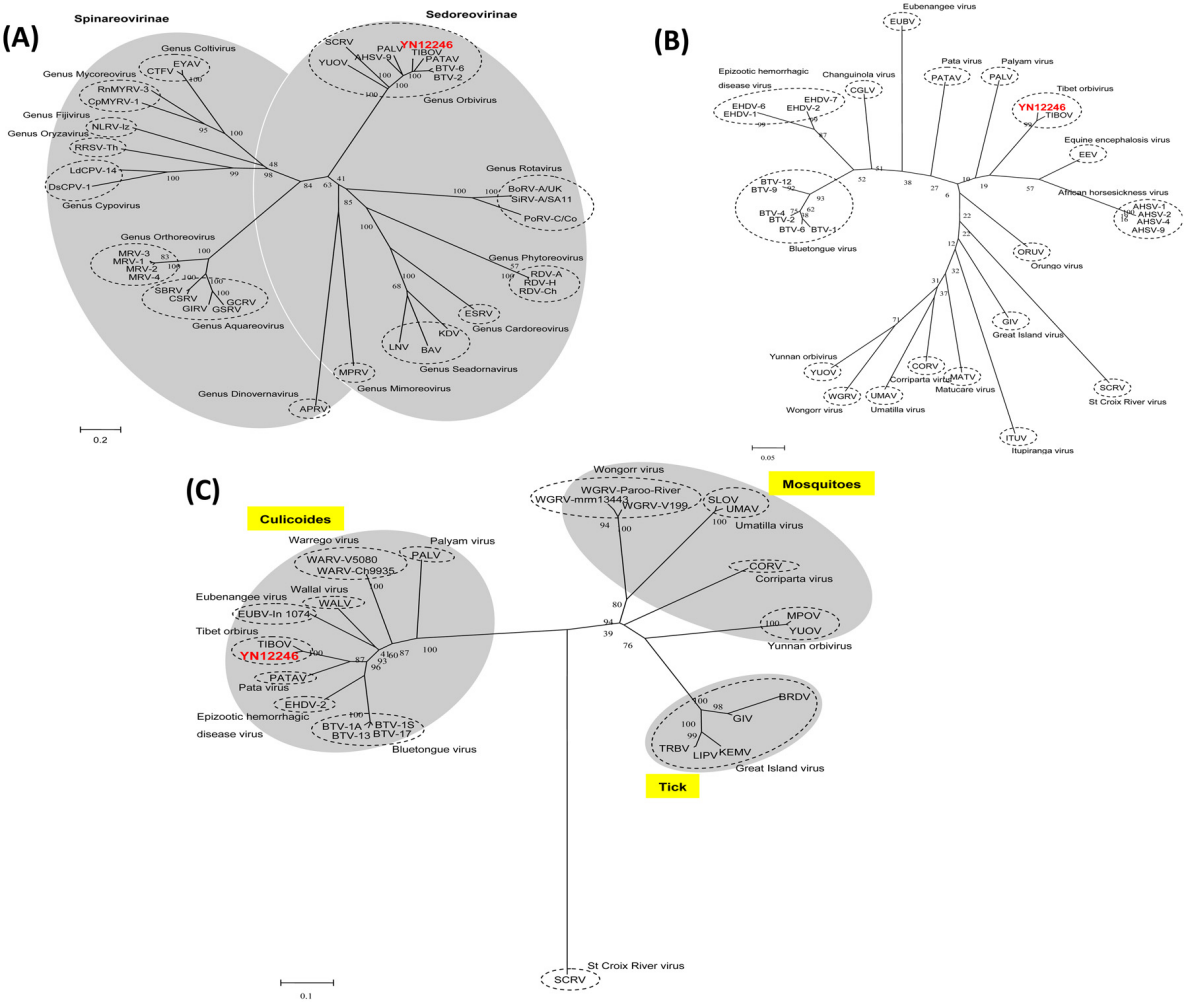
Phylogenetic analysis of VP1 amino acid sequences from (A) *Reoviridae* and (B) *Orbivirus* strains. (C) Phylogenetic analysis of T2 amino acid sequences from 26 *Orbivirus* strains. This analysis employed a neighbor-joining method (using the P-distance algorithm) using the MEGA software (version 5.05; www.megasoftware.net). Bootstrap probabilities for each node were calculated using 1000 replicates. Scale bars indicate the number of amino acid substitutions per site. In Fig 4C, because many of the available sequences are incomplete, the analysis was based on partial sequences (residues 356–567 relative to the BTV-1A sequence). Abbreviations and serotypes (or strain name) are shown in the radial tree image of Fig 4. GenBank accession numbers and further details of the sequences can be found in S1 Table (supplementary data).

To better understand the taxonomic classification of YN12246, the amino acid sequences of 37 VP1 proteins (S1 Table) covering 14 genera within the family *Reoviridae* were obtained from GenBank, and used to construct a phylogenetic tree. These 37 virus strains (including different species and different serotypes of one species) readily clustered into 14 evolutionary branches, with YN12246 clustering within the genus *Orbivirus* branch (Fig 4A). To further determine the genus of YN12246, VP1 amino acid sequences from 29 known orbivirus strains were used to construct a phylogenetic tree specific to this genus (S1 Table). This analysis demonstrated that YN12246 is most closely related to the TIBOV strain XZ0906, with the two strains clustering into the same phylogenetic branch (Fig 4B).

The gene encoding the major sub-core protein (T2) is the most conserved of the orbivirus genes. The T2 protein of *Orbivirus* is, therefore, useful for classifying members of the genus [8]. T2 amino acid sequences from 26 known orbivirus strains, along with the equivalent region from YN12246, were selected for construction of a phylogenetic tree. This analysis showed that YN12246 clustered into the same branch as the TIBOV strain XZ0906, suggesting that YN12246 from Yunnan is a strain of TIBOV (Fig 4C).

## Discussion

The parameters stipulated by the International Committee on Taxonomy of Viruses (ICTV) for the ‘polythetic definition’ of individual *Orbivirus* species include the reassortment of genome segments, genome segment migration patterns during 1% agarose gel electrophoresis (AGE), conserved terminal nucleotide sequences, serological cross-reactions, comparison of homologous genome segments or proteins by sequence analysis or cross-hybridization, host and vector range and the nature of clinical signs induced [3]. Recent advancements in molecular biology and sequencing technologies have allowed phylogenetic comparisons to be used as major tools for orbivirus identification, enabling taxonomic classification and the development of diagnostic tests. Sequence data generated for conserved orbivirus genes (e.g. the T2, polymerase or T13 protein genes) have been used previously for phylogenetic comparisons and taxonomic classification [18, 19].

The genetic sequences of VP1 and T2 can be used to define new species within the genus *Orbivirus*. The RdRp protein sequence, encoded by genome segment 1 (VP1), is an important criterion for the identification of members of genus *Orbivirus* [7, 8]. Previous studies had shown that all orbiviruses have more than 30% aa sequence identity with respect to viral RNA polymerase (VP1) [8]. The VP1 proteins from YN12246 and TIBOV XZ0906 share 98.8% aa identity. In contrast, YN12246 VP1 shares between 35.4% and 73% aa identity with VP1 from other representative *Orbivirus* members. These data indicate that YN12246 belongs to genus *Orbivirus*. In addition, the sub-core protein (T2) of *Orbivirus* is used to classify serotypes within the genus. It has also been demonstrated that orbiviruses within a single species group have more than 91% identity with respect to the T2 protein aa sequence [8]. YN12246 and TIBOV XZ0906 share 96.4% aa identity in relation to T2 protein. In contrast, a lower level of aa identity (23.3%- 78.3%) is seen between T2 protein from YN12246 and members of other *Orbivirus* species. These data indicate that YN12246 and XZ0906 belong to a single serotype. From the phylogenetic analysis of family *Reoviridae* and genus *Orbivirus*, based on the VP1 and T2 protein aa sequences, it is possible to conclude that YN12246 is a member of the TIBOV species.

Sequencing on the Ion Torrent PGM sequencer was completed, producing a total of 4,506,288 reads. A total of approximately 800,000 reads (data not shown) was obtained relative to the genus *Orbivirus* by bioinformatics analysis. RT-PCR amplification was used to close the gaps between contigs for each segment. Ultimately, the whole coding sequences of 8 segments of YN12246 (Seg1, Seg3-Seg5, Seg7-Seg10) and partial sequences of Seg6 (660–1633) were available. Then Sanger sequencing was employed to confirm the sequences using primers newly designed for each of the 9 RNA segments (Table 2). However, the sequences of Seg2 and partial sequences of Seg6 of YN12246 were not obtained. Although several contigs related to Seg2 and Seg6 of orbivirus were acquired, but RT-PCR or sequencing of PCR product all failed.

Previous studies have shown that the VP1 and VP3 proteins (encoded by Seg1 and 3 respectively) of BTV are highly conserved, while the components of the BTV outer capsid, proteins VP2 and VP5 (encoded by Seg2 and 6 respectively), are the most variable of the viral proteins. VP2, in particular, contains neutralising epitopes and controls the interactions between the virus particle and neutralising antibodies, exhibits a relatively low amino acid identity among the different BTV serotypes [20]. Amino acid identities between *Culicoides*–isolated strain (YN12246) and mosquito-isolated strain (XZ0906) were 98.8% for VP1 and 96.4% for VP3 (T2 protein) but only 63.6% within the VP6 gene (partial sequences), exhibited the lowest homology. On the other hand, the failure to obtain the sequence of Seg2 by high-throughput sequencing indicates a very low homology in the VP2 gene between *Culicoides*-isolated strain and known orbivirus. Accordingly, Seg2 may be the hypervariable region in TIBOV, further research will be carried out. We will continue to carry out the sequencing work of Seg2 and Seg6, in order to obtain the whole genome sequence of the virus YN12246.

The cumulative genetic information provided by many studies has confirmed that *Orbivirus* can be divided into three groups, namely *Culicoides*-borne orbiviruses (such as BTV), mosquito-borne orbiviruses (such as YUOV) and tick-borne orbiviruses (such as *Great Island virus*; GIV) [1, 19]. These studies not only demonstrated that orbiviruses are vector-borne viruses but also elucidated the propagation characteristics of various viruses, and identified evolutionary adaptations to the transmission vector. The result of the T2 protein phylogenetic analysis performed in this study is consistent with previous research. It is interesting to find that YN12246 isolated from *Culicoides* clusters onto the same branch as the TIBOV strain XZ0906 isolated from mosquitoes, in addition to BTV and EHDV, which are transmitted primarily by *Culicoides*, but is phylogenetically distinct from YUOV, UMAV (*Umatilla virus*) and SLOV (*Stretch Lagoon virus*), isolated from mosquitoes, as well as GIV, BRDV and LIPV (*Lipovnik virus*) isolated from ticks (Fig 4C). YN12246 and XZ0906 can both be clearly categorized into the *Culicoides*-borne group of viruses, suggesting that the TIBOV strains isolated from both mosquitoes and *Culicoides* can be considered *Culicoides*-borne orbiviruses. Although *Eubenangee virus* (EUBV), *Tilligery virus* (TILV), and PATAV were initially isolated from mosquitoes, EUBV has also been isolated from *Culicoides*, and the evolutionary relationship based on the analysis of the T2 subcore shell protein indicates that these three viruses group more closely with the *Culicoide*-borne orbiviruses, suggesting that *Culicoides* may act as their biological vector [21]. There is therefore precedent for orbiviruses originally isolated from one vector to be assigned to a phylogenetic group associated with a different vector.

Viruses within a single *Orbivirus* species/serogroup usually have similar dsRNA migration profiles in 1% agarose gel electrophoresis (AGE) [3]. The viral RNA migration band pattern, as resolved using PAGE, has been proposed as a criterion for the identification of *Orbivirus* [22]. YN12246, isolated from *Culicoides*, has a 3-3-3-1 RNA migration pattern almost identical to that of XZ0906, isolated from mosquitoes [9], suggesting that they belong to the same species. The RNA-PAGE migration pattern of YN12246 is broadly similar to those of EHDV, EUBV, TILV and PATAV [21], indicative of relatively close relationships between the different virus species. The PAGE migration data are therefore consistent with the T2 protein phylogenetic studies.

The indirect immunofluorescence assay (IFA) was applied to detect the IgG antibodies of swine serum samples collected in the virus isolation region against YN12246 strain. The results showed that a special fluorescent reaction appeared between YN12246 virus and swine serum, the antibody positive rate was 14.0% (8/57), while no positive for the serum samples collected from other regions was detected (data not shown). The results suggest that the TIBOV isolated from *Culicoides* was infectious to pigs, although the results of IgG antibody positive need to be verified by neutralization test.

Both TIBOV XZ0906, isolated from mosquitoes, and TIBOV YN12246, isolated from *Culicoides* and collected in Yunnan, could replicate in insect cells (C6/36) and mammalian cells (BHK-21). While TIBOV XZ0906 only caused CPE in BHK-21 cells, and not in C6/36 cells, TIBOV YN12246 caused CPE in both cell types. This result may reflect phenotypic differences between strains of one virus species isolated from different vectors. Further study is required to investigate this phenomenon further. In addition, although CPE progressed quickly after viral inoculation of cell cultures, with up to 75% of BHK-21 cells showing CPE after 3–4 days of YN12246 infection, multiple plaque formation revealed a relatively low virus titer. The titer of the TIBOV strain YN12246 was approximately 1.0 × 10^4^–1.5 × 10^4^ PFU/mL, similar to the XZ0906 strain (data not shown). This low virus titer might represent a defining characteristic of the TIBOV orbivirus.

## Supporting Information

S1 Table Details of all virus strains used in this study. (DOCX)
