# Correction: Isolation of *Tibet orbivirus*, TIBOV, from *Culicoides* Collected in Yunnan, China

**DOI:** 10.1371/journal.pone.0139646

**Published:** 2015-09-25

**Authors:** Wenwen Lei, Xiaofang Guo, Shihong Fu, Yun Feng, Kai Nie, Jingdong Song, Yang Li, Xuejun Ma, Guodong Liang, Hongning Zhou

There is an error in affiliation #3 for authors Xiaofan Guo and Hongning Zhou. Affiliation #3 should be: Yunnan Provincial Key Laboratory of Vector-borne Diseases Control and Research (in establishment) of Yunnan Institute of Parasitic Diseases.
