# Supplementary material for: Isolation of Tibet orbivirus, TIBOV, from Culicoides Collected in Yunnan, China
Source: PLoS One. 2015 Aug 21;10(8):e0136257. doi: 10.1371/journal.pone.0136257 (PMC4546636; doi:10.1371/journal.pone.0136257)
Supplement: S1 Table — (DOCX) [file pone.0136257.s001.docx]

**Supplementary Information**

**Table S1.** Details of all virus strains used in this study.

| **Genus** | **Species** | **Abbreviation** | **Strain/Serotype** | **GenBank accession No.** | |
| --- | --- | --- | --- | --- | --- |
|  |  |  |  | **VP1(RdRp)** | **T2** |
| *Orbivirus* | African horsesickness virus | AHSV-1 | HS29-62/serotype1 | FJ183364 | FJ183365 |
|  | African horsesickness virus | AHSV-2 | HS 02-07/serotype2 | FJ196584 | FJ196585 |
|  | African horsesickness virus | AHSV-4 | HS32-62/serotype4 | JQ796724 | JQ796725 |
|  | African horsesickness virus | AHSV-9 | E41-02(Or)/serotype9 | U94887 | DQ868776 |
|  | Bluetongue virus | BTV-1 | SZ97-1/serotype1 | JN848759 | JN848760 |
|  | Bluetongue virus | BTV-1A | Australia | NA | P20608 |
|  | Bluetongue virus | BTV-2 | BTV-2IT(L)/serotype2 | JN255862 | JN255863 |
|  | Bluetongue virus | BTV-4 | BTV-4IT(L)/serotype4 | JN255882 | JN255883 |
|  | Bluetongue virus | BTV-6 | USA2006-01/serotype6 | GQ506536 | GQ506538 |
|  | Bluetongue virus | BTV-9 | BTV-9IT(L)/serotype9 | JN255902 | JN255903 |
|  | Bluetongue virus | BTV-12 | BTV12-PT2003/serotype12 | GU390658 | GU390659 |
|  | Bluetongue virus | BTV-13 | USA | NA | Q65750 |
| Bluetongue virus  Bluetongue virus | BTV-1S  BTV-17 | South  USA | Africa  NA | NA  P03539 |  |
|  | Broadhaven virus | BRDV | BRDV | NA | P35934 |
|  | Changuinola virus | CGLV | BeAr478620 | HQ397615 | NA |
|  | Corriparta virus | CORV | CSIRO1740 | HQ397617 | NA |
|  | Corriparta virus | CORV | MRM1 | NA | AAM96695 |
|  | Epizootic hemorrhagic disease virus | EHDV-1 | New Jersey/serotype1 | NC_013396 | NC_013397 |
|  | Epizootic hemorrhagic disease virus | EHDV-2 | Ibaraki/serotype2 | AM745077 | AM745078 |
|  | Epizootic hemorrhagic disease virus | EHDV-6 | 318/serotype6 | AM745067 | AM745069 |
|  | Epizootic hemorrhagic disease virus | EHDV-7 | CSIRO 775/serotype7 | AM745047 | AM745048 |
|  | Equine encephalosis virus | EEV | HS103-06 | FJ183384 | FJ183385 |
|  | Eubenangee virus | EUBV | AUS1963/01 | JQ070376 | JQ070377 |
| Eubenangee virus | EUBV | In 1074 | NA | AF530087 |  |
| Great Island virus  Itupiranga virus | GIV  ITUV | CanAr-42  BeAr312086 | ADM88592  HQ397639 | ADM88593 |  |
|  |  |  |  | NA |  |
|  | Kemerovo virus | KEMV | EgAn 1169-61 | ADM88609 | ADM88610 |
|  | Lipovnik virus | LIPV | CzArLip-91 | ADM88603 | ADM88604 |
|  | Matucare virus | MATV | MARU21343 | HQ397640 | NA |
|  | Middle point orbivirus | MPOV | DPP4440 | ABU95014 | ABU95015 |
|  | Orungo virus | ORUV | IBH11306-84 | HQ397641 | NA |
|  | Palyam virus | PALV | Chuzan | BAA76549 | BAA34936 |
|  | Pata virus | PATAV | CAF1968/01 | JQ070386 | JQ070388 |
|  | St Croix River virus | SCRV | SCRV | AAG34363 | AAG34364 |
|  | Stretch Lagoon | SLOV | K49460 | ACH91290 | ACH91291 |
|  | Tibet orbivirus | TIBOV | XZ0906 | KF746187 | KF746189 |
|  | Tribec virus | TRBV | TRBV | ADM88606 | ADM88607 |
|  | Umatilla virus | UMAV | USA1969/01 | AEE98368 | AEE98369 |
|  | Wallal virus | WALV | Ch12048 | NA | AAM96693 |
|  | Warrego virus | WARV | V5080 | ABM92924 | ABM92926 |
|  | Warrego virus | WARV | Ch9935 | AAM96690 | AAM96692 |
|  | Wongorr virus | WGRV | CSIRO51 | HQ397668 | NA |
|  | Wongorr virus | WGRV | mrm13443 | NA | U56992 |
|  | Wongorr virus | WGRV | Paroo-River | NA | U56993 |
| Wongorr virus | WGRV | V199 | NA | U56991 |  |
|  | Yunnan orbivirus | YUOV | YOV-77-2 | YP443925 | YP443926 |
| *Genus Phytoreovirus* | Rice dwarf virus | RDV-A | A | BAA14222 | NA |
|  | Rice dwarf virus | RDV-Ch | Chinese | AAB18743 | NA |
|  | Rice dwarf virus | RDV-H | H | BAA01074 | NA |
| *Genus Rotavirus* | Rotavirus A (Bovine rotavirus A) | BoRV-A/UK | UK WT BRV4A | CAA39085 | NA |
|  | Rotavirus A (Bovine rotavirus A) | SiRV-A/SA11 | Simian | AAC58684 | NA |
|  | Rotavirus C (Bovine rotavirus C) | PoRV-C/Co | Co | AAB00801 | NA |
| *Genus Seadornavirus* | Banna virus | BAV | BAV-Ch | AAF77631 | NA |
|  | Kadipiro virus | KDV | JKT-7075 | AAF78848 | NA |
|  | Liao ning virus | LNV | LNSV-NE9731 | AAQ83562 | NA |
| *Genus Cardoreovirus* | Eriocheir sinensis reovirus | ESRV | 905 | AAT11887 | NA |
| *Genus Mimoreovirus* | Micromonas pusilla reovirus | MPRV | MPRV | AAZ94041 | NA |
| *Genus Aquareovirus* | Aquareovirus A (Chum salmon  reovirus) | CSRV | CSRV | AAL31497 | NA |
|  | Aquareovirus A (Striped bass reovirus) | SBRV | SBRV | AAM93410 | NA |
|  | Aquareovirus C (Grass carp reovirus) | GCRV | GCRV | AAG10436 | NA |
|  | Aquareovirus C (Golden shiner reovirus) | GSRV | GSRV | AAM92745 | NA |
|  | Aquareovirus G (Golden ide reovirus) | GIRV | GIRV | AAM93415 | NA |
| *Genus Cypovirus* | Dendrlymus punctatus cytoplas-mic polyhedrosis virus-1 | DsCPV-1 | DsCPV-1 | AAN46860 | NA |
|  | Lymantria dispar cytoplasmic polyhedrosis virus-14 | LdCPV-14 | LdCPV-14 | AAK73087 | NA |
| *Genus Coltivirus* | Colorado tick fever virus | CTFV | Florio | AAK00595 | NA |
|  | Eyach virus | EYAV | Fr578 | AAM18342 | NA |
| *Genus Dinovernavirus* | Aedes pseudoscutellaris reovirus | APRV | APRV | AAZ94068 | NA |
| *Genus Fijivirus* | Nilaparvata lugens reovirus | NLRV-Iz | Izumo | BAA08542 | NA |
| *Genus Mycoreovirus* | Mycoreovirus 1 (Cryphonectria parasitica reovirus) | CpMYRV-1 | 9B21 | AAP45577 | NA |
|  | Mycoreovirus 3 (Rosellinia anti-rot virus) | RnMYRV-3 | RArV | BAC98431 | NA |
| *Genus Orthoreovirus* | Mammalian orthoreovirus 1 | MRV-1 | Lang | AAA47234 | NA |
|  | Mammalian orthoreovirus 2 | MRV-2 | Jones | AAA47245 | NA |
|  | Mammalian orthoreovirus 3 | MRV-3 | Dearing | AAA47255 | NA |
|  | Mammalian orthoreovirus 4 | MRV-4 | Ndelle | AAL36027 | NA |
| *Genus Oryzavirus* | Rice ragged stunt virus | RRSV-Th | Thai | AAC36456 | NA |

**Note:** NA, Not available.
